# PI3K/Akt signaling pathway triggers P2X7 receptor expression as a pro-survival factor of neuroblastoma cells under limiting growth conditions

**DOI:** 10.1038/srep18417

**Published:** 2015-12-21

**Authors:** Rosa Gómez-Villafuertes, Paula García-Huerta, Juan Ignacio Díaz-Hernández, Mª Teresa Miras-Portugal

**Affiliations:** 1 Departamento de Bioquímica y Biología Molecular IV, Facultad de Veterinaria, Universidad Complutense de Madrid, 28040 Madrid, Spain; 2 Instituto de Investigación Sanitaria del Hospital Clínico San Carlos, Madrid, Spain

## Abstract

The expression of purinergic P2X7 receptor (P2X7R) in neuroblastoma cells is associated to accelerated growth rate, angiogenesis, metastasis and poor prognosis. Noticeably, P2X7R allows the survival of neuroblastoma cells under restrictive conditions, including serum and glucose deprivation. Previously we identified specificity protein 1 (Sp1) as the main factor involved in the transcriptional regulation of *P2rx7* gene, reporting that serum withdrawal triggers the expression of P2X7R in Neuro-2a (N2a) neuroblastoma cell line. Here we demonstrate that PI3K/Akt pathway is crucial for the upregulation of P2X7R expression in serum-deprived neuroblastoma cells, circumstance that facilitates cell proliferation in the absence of trophic support. The effect exerted by PI3K/Akt is independent of both mTOR and GSK3, but requires the activation of EGF receptor (EGFR). Nuclear levels of Sp1 are strongly reduced by inhibition of PI3K/Akt pathway, and blockade of Sp1-dependent transcription with mithramycin A prevents upregulation of *P2rx7* gene expression following serum withdrawal. Furthermore, atypical PKCζ plays a key role in the regulation of P2X7R expression by preventing phosphorylation and, consequently, activation of Akt. Altogether, these data indicate that activation of EGFR enhanced the expression of P2X7R in neuroblastoma cells lacking trophic support, being PI3K/Akt/PKCζ signaling pathway and Sp1 mediating this pro-survival outcome.

Nucleotides are an ubiquitous family of signaling molecules that exert different extracellular effects through interaction with two families of purinergic receptors: G-protein coupled P2Y receptors and ligand-gated P2X cation channels. So far, seven P2X subunits (P2X1-7) and eight P2Y receptors (P2Y_1,2,4,6,11,12,13,14_) have been cloned and characterized according to their agonist sensitivity, sequence identities and signal transduction mechanism. There is a growing interest in the therapeutic potential of nucleotide receptors for the treatment of cancer^1^. Extracellular ATP, an abundant component of the tumor microenvironment, is emerging as a new and potent regulator of cancer progression and immune response modulator^2^,^3^. Intriguingly, whereas high doses of ATP have a strong cytotoxic effect on several tumors, lower ATP concentrations, reached after spontaneous release of this nucleotide from cells, have a growth-promoting effect^4^. Among purinergic receptors, P2X7 seems to be the best candidate to confer a growth advantage to cancer cells *in vivo*^5^. This receptor is highly expressed by nearly all human cancers so far investigated^1^,^4^, including neuroblastoma cells from both primary tumors and cell lines^6^. Neuroblastoma is a neuroendocrine tumor, responsible for 15% of pediatric cancer deaths. They may originate in any part of the sympathetic nervous system, most commonly in the adrenal medulla and sympathetic ganglia, and secondary tumors are often widespread in other organs and bone^7^. Neuroblastoma progression is frequently associated with high rates of proliferation even in the absence of trophic support, and several studies have demonstrated that the degree of tumor differentiation influences patient outcome^7^. Therefore, a better understanding of the genes, proteins, and pathways responsible for neuroblastoma tumorigenesis and progression may lead to the development of more effective, less toxic therapies. In contrast to many other non-tumor cell types, *in vitro* stimulation of P2X7R does not induce caspase-3 activation or apoptosis of neuroblastoma cells, but rather supported their survival and proliferation in the absence of serum by triggering the release of trophic factors^4^,^6^. Recent findings provide direct *in vivo* evidences that tumors engineered to overexpress P2X7R show accelerated *in vivo* growth rate, enhanced angiogenesis and increased tendency to metastasize, whereas P2X7R inhibition slows down tumor progression^5^,^8^. Moreover, the analysis of P2X7 expression in a patient’s cohort revealed that high P2X7 levels correlates with poor prognosis of stage IV neuroblastoma patients^9^. In previous studies we characterized that P2X7R silencing or pharmacologic blockade led to an increase in neurite formation in murine N2a neuroblastoma cells through a Ca^2+^-calmodulin dependent kinase II signaling cascade, and that P2X7R is involved in the maintenance of neuroblastoma cells in a non-differentiated state^10^. A parallel study also showed that a decrease in the expression of P2X7R is associated with neuronal differentiation and that P2X7R activation is important in maintaining cell survival of neuroblastoma cells^11^. Using a chimeric plasma membrane-targeted luciferase, which allows *in vivo* measurement of extracellular ATP, hundred micromolar concentration of this nucleotide has been specifically detected in neuroblastoma tumor microenvironment, while it is basically undetectable in healthy tissues^12^,^13^. Moreover, we have reported a positive feedback mechanism mediated by P2X7R-stimulated exocytotic release of ATP that would activate P2X7Rs from the same or neighboring neuroblastoma cells to further stimulate its own release and negatively control cell differentiation^14^. The trophic signaling cascade activated by P2X7R involves a strong enhancement in the efficiency of mitochondrial oxidative phosphorylation, a higher cellular ATP level, an increased Ca^2+^ content of the endoplasmic reticulum, and an activation of NFATc1, a key transcription factor in cancer cell growth^15^,^16^. Moreover, during glucose deprivation P2X7R overexpression correlates with a higher lactate output, overexpression of several glycolytic enzymes and larger intracellular glycogen stores, allowing better adaptability to unfavorable ambient conditions^17^. Based on these findings, a deeper understanding of the relationship between trophic deprivation and P2X7R expression could be biologically and clinically important. We have previously investigated the mechanisms underlying transcriptional regulation of P2X7R in N2a neuroblastoma cells, identifying Sp1 as the main transcription factor involved in the regulation of *P2rx7* gene^18^. Moreover, we evidenced that serum withdrawal was able to increase the expression of P2X7 transcript in neuroblastoma cells, although the mechanism implicated remained unknown. The purpose of this study was to elucidate the signaling pathways underlying the transcriptional upregulation of *P2rx7* gene expression in neuroblastoma cells following serum starvation. We report here that serum deprivation triggers EGFR-dependent activation of PI3K/Akt pathway, which is crucial for the upregulation of *P2rx7* gene expression via Sp1 factor. Moreover, atypical PKCζ is a key component in the regulation of *P2rx7* gene expression through the inactivation of Akt. We also demonstrated that the increase in P2X7R expression induced by serum withdrawal in N2a cells is a pro-survival mechanism to compensate the lack of trophic support.

## Results

### Upregulation of P2X7R following serum withdrawal facilitates neuroblastoma cells proliferation

**Figure 1:**
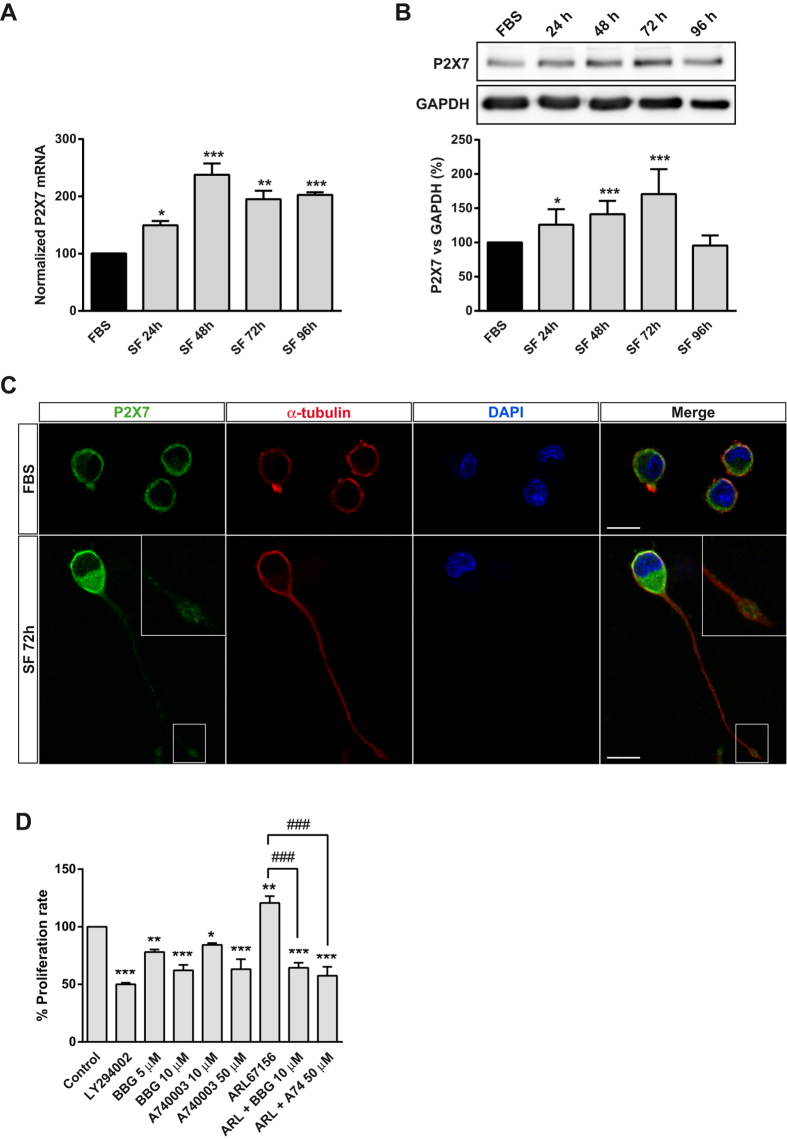
P2X7R expression is upregulated in serum-deprived neuroblastoma cells. (**A**) Changes in P2X7 transcript levels in N2a cell line cultured either in standard culture medium (FBS) for 24 h or in serum free medium (SF) for the indicated time periods. Total RNA was extracted and P2X7 mRNA was quantified by Q-PCR as described in M*ethods*. GAPDH was used as a control for differences in cDNA input. Results are mean ± s.e.m. of three independent experiments in triplicate; *P ≤ 0.05, **P ≤ 0.01, ***P ≤ 0.001 *vs* FBS (ANOVA with the Dunnett’s post hoc test). (**B**) Immunoblotting depicting the presence of endogenous P2X7R in whole-cell lysates from N2a cells cultured either in FBS for 24 or in SF at the indicated time points. Cell lysates were analyzed by western blotting with anti-P2X7R (intracellular epitope) antibody. GAPDH was used as internal loading control. Histogram represents P2X7 protein levels at the indicated time periods obtained by densitometry and normalization to GAPDH. The values represent mean ± s.e.m. of three independent experiments in duplicate. *P ≤ 0.05, ***P ≤ 0.001 *vs* FBS (ANOVA with the Dunnett’s post hoc test). (**C**) Double immunofluorescence for P2X7R (green) and α-tubulin (red) in N2a cells cultured either in FBS for 24 h or in SF medium for 72 h. Nuclei were counterstained with DAPI (blue). Insets depict enlarged views (2.5X magnification) of delimited area. Scale bar = 15 μm. (**D**) Proliferation of serum-starved N2a cells treated with LY294002 (50 μM), BBG (5 or 10 μM), A740003 (10 or 50 μM) and/or ARL67156 (100 μM) for 48 h. Values were normalized to those obtained from untreated control cells, set as 100% proliferation rate. Results are mean ± s.e.m. of three independent experiments in triplicate. *P ≤ 0.05, **P ≤ 0.01, ***P ≤ 0.001 *vs* control (ANOVA with the Dunnett’s post hoc test); ^###^P < 0.001 (ANOVA with the Sidak’s post hoc test).

In order to confirm that serum deprivation enhances P2X7R expression, N2a cells were cultured in either DMEM supplemented with 10% FBS (FBS) or in serum-free medium (SF) for 24, 48, 72, and 96 h. Quantitative real-time PCR (Q-PCR) analysis revealed that N2a cells cultured in SF underwent a strong increment of P2X7 transcript (Fig. 1A). Western blot and immunofluorescence studies corroborated that P2X7 protein levels were increased up to 70% in cells maintained in SF for 72 h (Fig. 1B,C, respectively). In an effort to evaluate whether the upregulation of P2X7R following serum withdrawal also occurs in a human cellular model, SH-SY5Y neuroblastoma cell-line expressing functional P2X7R were used^19^. As expected, Q-PCR studies indicated that SH-SY5Y cells cultured in SF suffer an increment in P2X7 transcript comparable to that observed in murine neuroblastoma cells (see supplementary Fig. S1A online). Based on previous studies reporting that P2X7R supports proliferation of neuroblastoma cells in the absence of serum by triggering the release of trophic factors^4^,^6^, we postulated that the upregulation of P2X7R following serum deprivation could be facilitating neuroblastoma cells survival. To evaluate this possibility, we measured N2a cell growth in SF medium following administration of two different P2X7R antagonists, Brilliant blue G (BBG) and the competitive antagonist A740003^10^,^20^; as well as the inhibitor of ecto-ATPases ARL67156 (100 μM)^14^. After 48 h, both BBG and A740003 caused a dose-dependent reduction of cell proliferation, even comparable to that induced by the PI3K inhibitor LY294002 (50 μM), which is a central player of neuroblastoma cell growth and progression^21^. Moreover, inhibition of ecto-ATPase activity, which enhances extracellular ATP concentration, resulted in a higher proliferation rate, effect that was completely abolished by either BBG or A740003 (Fig. 1D).

**Figure 2:**
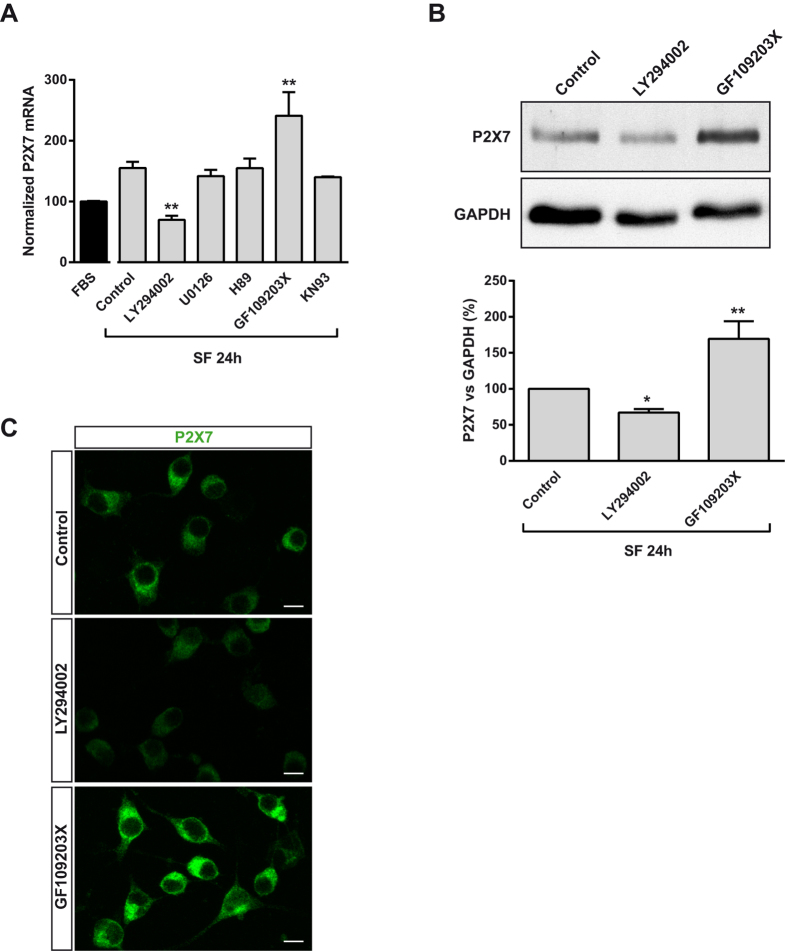
Involvement of PI3K and PKC in the regulation of P2X7R expression in neuroblastoma cells following serum withdrawal. (**A**) N2a cells were incubated in FBS medium for 24 h or in SF medium in absence (control) or presence of LY294002 (50 μM, PI3K inhibitor), U0126 (10 μM, MEK/ERK1/2 inhibitor), H89 (1 μM, PKA inhibitor), GF109203X (5 μM, pan PKC inhibitor) or KN93 (1 μM, CaMKII inhibitor) in SF medium for 24 h. Total RNA was extracted and P2X7 mRNA was quantified by Q-PCR, using GADPH as housekeeping gene. Normalized P2X7 transcript levels in cells cultured in FBS was set as 100%. Results are mean ± s.e.m. of three independent experiments in triplicate. **P ≤ 0.01 *vs* control (ANOVA with the Dunnett’s post hoc test). (**B**) Immunoblotting showing the presence of endogenous P2X7R in N2a cells cultured in SF medium for 24 h in the absence (control) or presence of either LY294002 or GF109203X. Whole-cell lysates were analyzed by western blotting with anti-P2X7R antibody. GAPDH was used as internal loading control. Histogram represents levels of P2X7 protein in control cells compared to treated cells and were obtained by densitometry and normalization to GAPDH. Values are mean ± s.e.m. of three independent experiments in duplicate; *P ≤ 0.05, **P ≤ 0.01 *vs* control (ANOVA with the Dunnett’s post hoc test). (**C**) Immunofluorescence for P2X7R (green) in N2a cells cultured in SF medium for 24 h in the absence (control) or presence of either LY294002 or GF109203X. Scale bar = 15 μm.

Next, in order to elucidate the signaling pathway involved in the upregulation of P2X7R exerted by serum withdrawal, N2a cells were switched to SF medium for 24 h in the presence or absence of specific inhibitors of a variety of cytosolic protein kinases. The PI3K inhibitor LY294002 (50 μM) strongly reduced the expression of P2X7 transcript in cells cultured in SF (Fig. 2A). In contrast, treatment with a pan PKC inhibitor, GF109203X (10 μM), significantly increased P2X7 mRNA levels compared to non-treated cells. Finally, the MEK inhibitor U0126 (10 μM), the PKA inhibitor H89 (1 μM) or the CaMKII inhibitor KN93 (1 μM) had no effect. Western blot and immunofluorescence studies confirmed that LY294002 prevented the upregulation of P2X7R expression observed in serum-deprived neuroblastoma cells, whereas GF109203X exerted the opposite effect (Fig. 2B,C, respectively).

### PI3K/Akt pathway regulates P2X7R expression in neuroblastoma cells independently of GSK3 and mTOR

**Figure 3:**
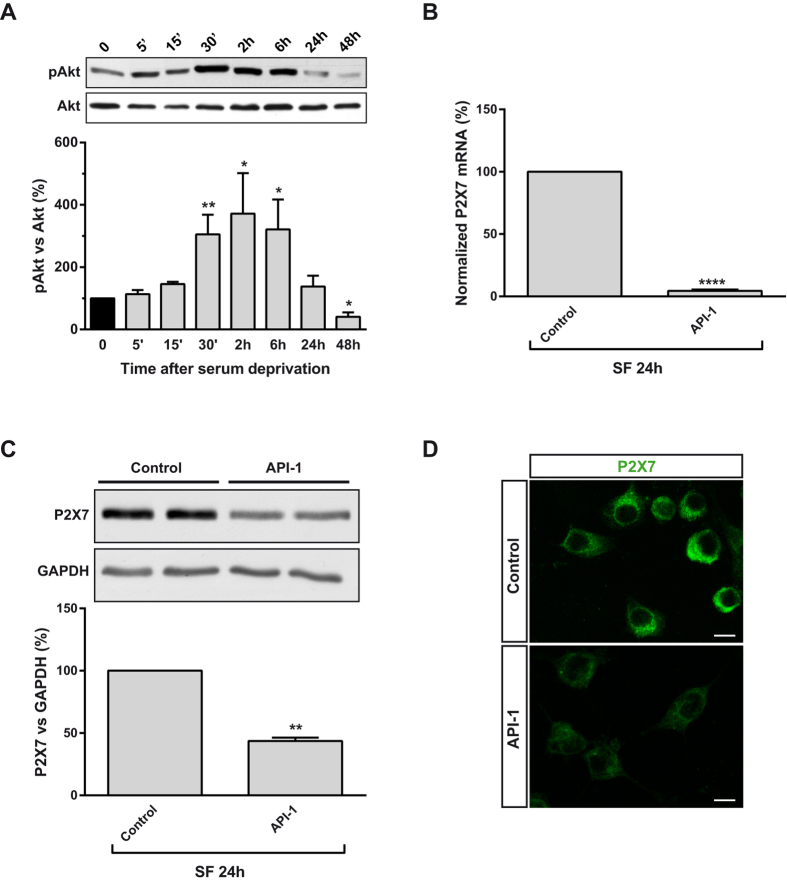
Akt activation induced by serum withdrawal triggers upregulation of P2X7R receptor expression in neuroblastoma cells. (**A**) Changes in Akt phosphorylation in N2a cells cultured in SF medium for the indicated time points. Whole-cell lysates were analyzed by western blotting with antibodies against phospho-Akt (Thr^308^) or total Akt. Histogram represents relative levels of phospho-Akt (pAkt) during the whole detection period, obtained by densitometry and normalization to total Akt. The values represent mean ± s.e.m. of four independent experiments in duplicate. *P ≤ 0.05, **P ≤ 0.01 *vs* time = 0 (ANOVA with the Dunnett’s post hoc test). (**B**) N2a cells were incubated in absence or presence of API-1 (10 μM, Akt inhibitor) in SF medium for 24 h. Total RNA was extracted and P2X7 mRNA was quantified by Q-PCR, using GADPH as housekeeping gene. Results are mean ± s.e.m. of three independent experiments in triplicate. ***P ≤ 0.001 *vs* control (t-test). (**C**) Immunoblotting depicting the presence of endogenous P2X7R in whole-cell lysates from N2a cells cultured in SF medium for 24 h in the absence (control) or presence of API-1. Whole-cell lysates were analyzed by western blotting with anti-P2X7R antibody. GAPDH was used as internal loading control. Histogram represents levels of P2X7 protein in control cells compared to treated cells, obtained by densitometry and normalization to GAPDH. Values are mean ± s.e.m. of three independent experiments in duplicate; **P ≤ 0.01 *vs* control (t-test). (**D**) Immunofluorescence for P2X7R (green) in N2a cells cultured in SF medium for 24 h in the absence (control) or presence of API-1. Scale bar = 15 μm.

As previously mentioned, PI3K-dependent signaling seems to be one of the most potent pro-survival pathways involved in neuroblastoma tumorigenesis^22^,^23^. Clinical mediators of PI3K include Akt, which is known to enhance cellular survival by stimulating cell proliferation and angiogenesis, and by suppressing apoptosis^24^,^25^. Serum deprivation induced a prolonged phosphorylation of Akt activation loop (Thr^308^) in N2a cells, reaching plateau levels 30 min after serum starvation and recovering control values (t = 0) 24 h later (Fig. 3A). To test whether there was a correlation between serum withdrawal-dependent phosphorylation of Akt and upregulation of *P2rx7* gene expression, an Akt inhibitor (API-1) was assayed. Treatment of N2a cells cultured in SF for 24 h with 10 μM API-1 nearly abolished the expression of P2X7 transcript (Fig. 3B). Western blot and immunofluorescence studies confirmed that API-1 drastically reduced P2X7R levels in cells cultured in the absence of serum (Fig. 3C,D, respectively). The effect of PI3K/Akt inhibition on P2X7R expression was also confirmed in a human cell line. Thus, treatment of serum-deprived SH-SY5Y cells with either LY29004 or API-1 for 24 h significantly reduced P2X7 transcript levels, indicating that PI3K/Akt pathway seems to have a key role in the regulation of P2X7R in neuroblastoma cells (see supplementary Fig. S1B online). At least 13 Akt substrates have been identified in mammalian cells, including regulators of apoptosis, cell growth, and cell cycle^24^. The main downstream targets of Akt involved in cell proliferation are GSK3 and the mammalian target of rapamycin (mTOR). To investigate their participation in the regulation of P2X7 expression, N2a cells were switched to SF for 24 h in the presence or absence of either the mTOR inhibitor rapamycin (2.5 nM) or the GSK3 inhibitor SB216763 (5 μM). Noticeable, neither rapamycin nor SB216763 modified P2X7 transcript levels (see supplementary Fig. S2A online). To further verified that GSK3 is not involved in the regulatory effect exerted by Akt, N2a cells were transiently transfected with either wild-type GSK3β (wtGSK3) or a constitutively active GSK3β mutant (GSK3^S9A^) in which serine 9 was mutated to alanine^26^. Overexpression of wtGSK3 or GSK3^S9A^ did not change the levels of either P2X7 transcript or protein compared to control cells transfected with empty vector (see supplementary Fig. S2B and C online, respectively). Taken together, these findings indicate that the transcriptional upregulation of *P2rx7* gene expression induced by serum withdrawal is independent of mTOR and GSK3.

### PI3K/Akt increased *P2rx7* gene expression via Sp1

**Figure 4:**
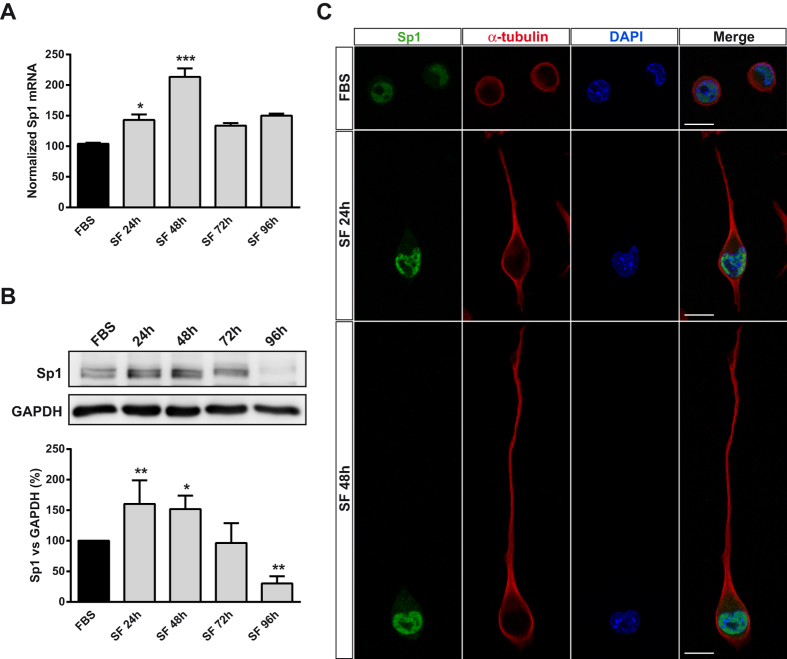
Sp1 transcription factor is upregulated in serum-deprived neuroblastoma cells. (**A**) Changes in Sp1 transcript levels in N2a cells cultured either in FBS medium for 24 h or in SF medium for the indicated time periods. Total RNA was extracted and Sp1 mRNA was quantified by Q-PCR. GAPDH was used as a control for differences in cDNA input. Results are mean ± s.e.m. of three independent experiments in triplicate. *P ≤ 0.05, ***P ≤ 0.001 *vs* FBS (ANOVA with the Dunnett’s post hoc test). (**B**) Immunoblotting depicting the presence of endogenous Sp1 in whole-cell lysates from N2a cells cultured in FBS for 24 or in SF at different time points. Cell lysates were analyzed by western blotting with anti-Sp1 antibody. GAPDH was used as an internal loading control. Histogram represents total Sp1 levels during the whole detection period, obtained by densitometry and normalization to GAPDH. The values represent mean ± s.e.m. of three independent experiments in duplicate. *P ≤ 0.05, **P ≤ 0.01 *vs* FBS (ANOVA with the Dunnett’s post hoc test). (**C**) Double immunofluorescence for Sp1 (green) and α-tubulin (red) in N2a cells cultured either in FBS for 24 h or in SF medium for 72 h. Nuclei were counterstained with DAPI (blue). Scale bar = 15 μm.

**Figure 5:**
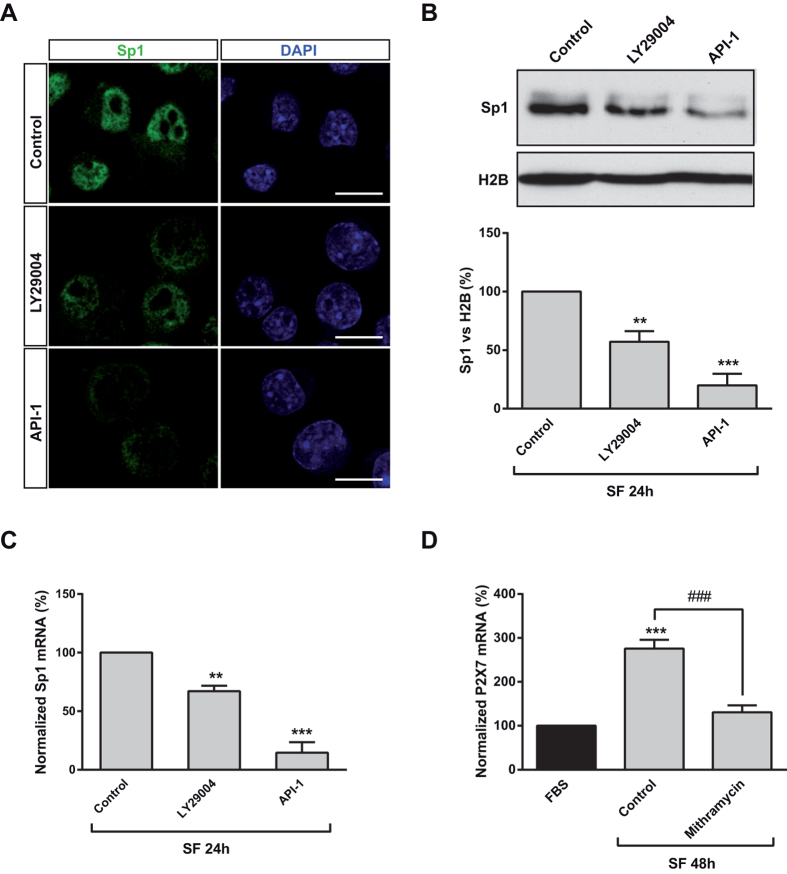
PI3K/Akt-dependent upregulation of P2X7 expression in serum-deprived neuroblastoma cells requires Sp1 factor. (**A**) Immunofluorescence for Sp1 (green) in N2a cells cultured in SF medium for 24 h in the absence (control) or presence of either LY294002 or API-1. Scale bar = 15 μm. (**B**) Immunoblotting depicting the presence of endogenous Sp1 in nuclear fractions of control and either LY294002- or API-1-treated N2a cells cultured in SF medium for 24 h. Histone H2B was used as nuclear fraction marker. Histogram represents levels of Sp1 protein in control cells compared to treated cells, obtained by densitometry and normalization to H2B. The values represent mean ± s.e.m. of four independent experiments in duplicate. **P ≤ 0.01, ***P ≤ 0.001 *vs* control (ANOVA with the Dunnett’s post hoc test). (**C**) Serum-deprived N2a cells were incubated in absence or presence of either LY294002 or API-1 for 24 h. Total RNA was extracted and Sp1 transcript was quantified by Q-PCR, using GADPH as housekeeping gene. Results are mean ± s.e.m. of three independent experiments in triplicate. **P ≤ 0.01, ***P ≤ 0.001 *vs* control (ANOVA with the Dunnett’s post hoc test). (**D**) Changes in P2X7 transcript levels in N2a cells cultured either in FBS for 24 h or in SF medium for 48 h in absence (control) or presence of mithramycin A (300 nM, Sp1 inhibitor). Total RNA was extracted and P2X7 mRNA was quantified by Q-PCR, using GADPH as a housekeeping gene. Normalized P2X7 transcript levels in cells cultured in FBS was set as 100%. Results are mean ± s.e.m. of three independent experiments in triplicate; ***P ≤ 0.001 *vs* FBS (ANOVA with the Dunnett’s post hoc test); ^###^P < 0.001 (ANOVA with the Sidak’s post hoc test).

In a previous study, we reported that Sp1 plays a crucial role in the transcriptional regulation of *P2rx7* gene^18^. To examine whether PI3K/Akt-dependent upregulation of *P2rx7* expression was mediated by Sp1, N2a cells were cultured in either FBS or SF medium and Sp1 mRNA levels were quantified at different time periods. After 24 h, a significant upregulation of Sp1 transcript was observed, being even more evident 48 h later and decreasing to basal levels thereafter (Fig. 4A). This observation is easily explained because Sp1 is an autoregulated transcription factor, and an increase in the transcriptional activity of Sp1 would lead to an increase in *Sp1* gene expression^27^. The effect of serum deprivation on Sp1 expression was confirmed by Western blot and immunofluorescence studies, both showing that Sp1 protein was upregulated in cells cultured in SF (Fig. 4B,C, respectively). The stability, location and transcriptional activity of Sp1 is precisely controlled by post-transcriptional modifications, including phosphorylation by protein kinases such as Akt^28^. Treatment of serum deprived N2a cells with either LY29004 or API-1 for 24 h significantly reduced nuclear levels of Sp1 (Fig. 5A,B), as well as Sp1 transcript (Fig. 5C), due to the auto-regulatory property of Sp1 previously mentioned. Moreover, inhibition of PI3K also decreased Sp1 protein levels in SH-SY5Y human neuroblastoma cells (see supplementary Fig. S1C), pointing to PI3K as the main regulator of Sp1 transcriptional activity in neuroblastoma cells. In order to confirm that Sp1 factor plays a critical role in the upregulation of P2X7 transcript exerted by serum deprivation, mithramycin A, an inhibitor of Sp1-dependent transcriptional activity, was assayed. Treatment of serum-deprived cells with 300 nM mithramycin for 48 h completely blocked the upregulation of *P2rx7* gene expression observed in serum-deprived N2a cells (Fig. 5D). Altogether, these results reveal that serum deprivation induces activation of PI3K/Akt pathway that results in Sp1-induced expression of *P2rx7* gene in neuroblastoma cells.

### Upregulation of P2X7R upon serum withdrawal requires the activation of EGFR

**Figure 6:**
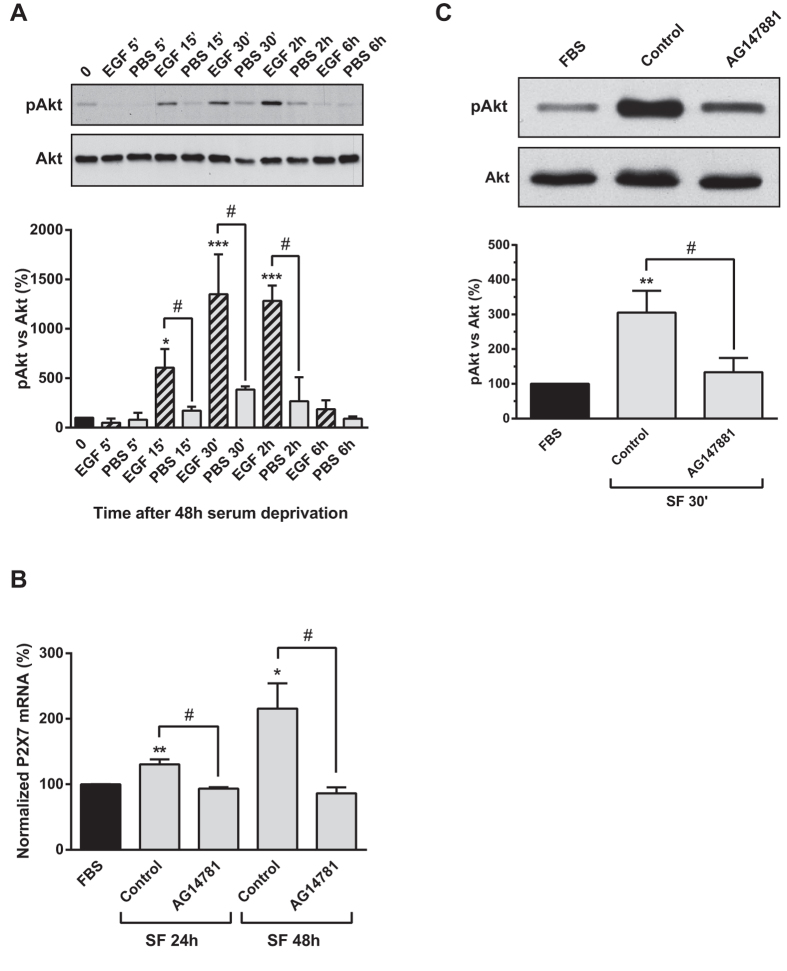
Akt phosphorylation induced by serum withdrawal in neuroblastoma cells is dependent on EGFR activation. (**A**) Serum-starved N2a cells were challenged with either vehicle (PBS) or EGF (100 ng/mL) for the indicated time periods. The expression of phospho-Akt (Thr^308^) and total Akt were detected by immunoblotting of whole-cell extracts. Histogram represents relative levels of phospho-Akt (pAkt) during the whole detection period, obtained by densitometry and normalization to total Akt. The values represent mean ± s.e.m. of four independent experiments in triplicate. *P ≤ 0.05, ***P ≤ 0.001 *vs* time = 0 (ANOVA with the Dunnett’s post hoc test); ^#^P < 0.05 (ANOVA with the Sidak’s post hoc test). (**B**) Serum-starved N2a cells were incubated either in FBS for 24 h or in SF for 24 h or 48 h in absence (control) or presence of AG147801 (1 μM, EGFR antagonist). Then, total RNA was extracted and P2X7 mRNA was quantified by Q-PCR, using GADPH as a housekeeping gene. Normalized P2X7 transcript levels in cells cultured in FBS medium for 24 h was set as 100%. Results are mean ± s.e.m. of three independent experiments in triplicate; *P ≤ 0.05, **P ≤ 0.01 *vs* FBS (ANOVA with the Dunnett’s post hoc test); ^#^P < 0.05 (ANOVA with the Sidak’s post hoc test). (**C**) Changes in Akt phosphorylation in N2a cells cultured in either FBS or in SF medium for 30 min in absence (control) or presence of AG147801. Whole-cell lysates were analyzed by western blotting with antibodies against phospho-Akt (Thr^308^) or total Akt. Histogram represents relative levels of phospho-Akt (pAkt) obtained by densitometry and normalization to total Akt. The values represent mean ± s.e.m. of three independent experiments in duplicate. **P ≤ 0.01 *vs* FBS (ANOVA with the Dunnett’s post hoc test); ^#^P < 0.05 (ANOVA with the Sidak’s post hoc test).

Activation of PI3K/Akt signaling pathway is typically found downstream of tyrosine kinase receptors. Previous studies have shown that serum deprivation triggers ligand-independent catalytic auto-activation of EGFR in N2a cells^29^. In order to confirm that N2a cells express functional EGFR coupled to activation of PI3K/Akt pathway, cells were cultured in SF medium for 48 h to reach basal phospho-Akt levels, and then stimulated with EGF (100 ng/mL) for several time periods. As shown in Fig. 6A, EGF mimicked the effect of serum starvation, since it was able to induce Akt phosphorylation (Thr^308^), reaching plateau levels 30 min after administration. To check whether EGFR activation was required for the transcriptional upregulation of *P2rx7* gene after serum starvation, cells were treated with AG14781 (1 μM), an inhibitor of EGFR tyrosine kinase. Interestingly, EGFR inactivation completely blocked the increase in P2X7 transcript observed upon serum starvation (Fig. 6B). Moreover, EGFR inhibition also reduced Akt phosphorylation induced by serum deprivation (Fig. 6C), indicating that EGFR activation is necessary to trigger PI3K/Akt signaling pathway and, consequently, to induce Sp1-dependent upregulation of P2X7R in neuroblastoma cells lacking trophic support.

### Atypical PKCζ decreased *P2rx7* gene expression by preventing Akt activation

**Figure 7:**
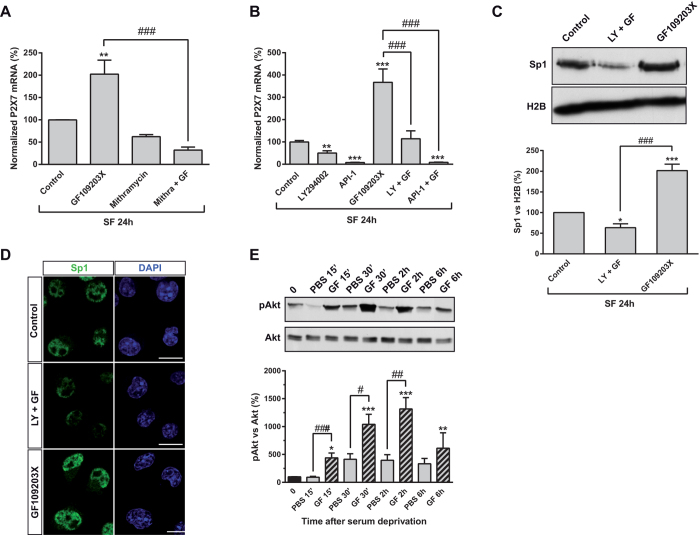
PKCζ decreased *P2rx7* gene expression by preventing Akt activation in serum deprived neuroblastoma cells. (**A**) Changes in P2X7 transcript levels in serum-deprived N2a cells cultured for 48 h in absence or presence of 10 μM GF109203X and/or 300 nM mithramycin A (Sp1 inhibitor). GADPH was used as housekeeping gene. Results are mean ± s.e.m. of three independent experiments in triplicate; **P ≤ 0.01 *vs* control (ANOVA with the Dunnett’s post hoc test); ^###^*P* ≤ 0.001 (ANOVA with the Sidak’s post hoc test). (**B**) Serum-deprived N2a cells were incubated for 24 h in absence (control) or presence of LY294002 (50 μM), API-1 (10 μM) and/or GF109203X (10 μM). P2X7 mRNA was quantified by Q-PCR, using GADPH as housekeeping gene. Results are mean ± s.e.m. of three independent experiments in triplicate. **P ≤ 0.01, ***P ≤ 0.001 *vs* control (ANOVA with the Dunnett’s post hoc test); ^###^*P* ≤ 0.001 (ANOVA with the Sidak’s post hoc test). (**C**) Immunoblotting depicting the presence of Sp1 in nuclear fractions of N2a cells cultured for 24 h in SF medium in absence (control) or presence of LY294002 (50 μM) and/or GF109203X (10 μM). Histone H2B was used as nuclear fraction marker. Histogram represents levels of Sp1 protein obtained by densitometry and normalization to H2B expression. The values represent mean ± s.e.m. of three independent experiments in duplicate. *P ≤ 0.05, ***P ≤ 0.001 *vs* control (ANOVA with the Dunnett’s post hoc test); ^###^*P* ≤ 0.001 (ANOVA with the Sidak’s post hoc test). (**D**) Immunofluorescence for Sp1 (green) in N2a cells cultured in SF medium for 24 h in the absence (control) or presence of LY294002 and/or GF109203X. Scale bar = 15 μm. (**E**) Serum-starved N2a cells were challenged with either vehicle (PBS) or GF109203X for the indicated time periods. The expression of phospho-Akt (Thr^308^) and total Akt were detected by immunoblotting of whole-cell extracts. Histogram represents relative levels of phospho-Akt (pAkt) obtained by densitometry and normalization to total Akt levels. The values represent mean ± s.e.m. of four independent experiments in duplicate. *P ≤ 0.05, **P ≤ 0.01, ***P ≤ 0.001 *vs* t = 0 (ANOVA with the Dunnett’s post hoc test); ^#^*P* ≤ 0.05, ^##^*P* ≤ 0.01, ^###^*P* ≤ 0.001 (ANOVA with the Sidak’s post hoc test).

The protein kinase C (PKC) family is a group of serine/threonine protein kinases that consists of at least 10 members divided into three subfamilies according to their structural and regulatory properties: classical PKCs (cPKCs) that are activated by both diacylglycerol (DAG) and Ca^2+^, novel PKCs (nPKCs) that do not respond to Ca^2+^ but require DAG, and atypical PKCs (aPKCs) that are activated by neither DAG nor Ca^2+^ 30. As previously showed, treatment of N2a cells with the pan PKC inhibitor 10 μM GF109203X resulted in a strong upregulation of *P2rx7* gene expression compared to untreated cells (Fig. 2A). To shed light on the subtype of PKC involved in this effect, serum-deprived cells were treated for 24 h with two different concentrations of GF109203X: 1 μM, which only effectively inhibits cPKCs and nPKCs, and 10 μM, which inhibits all PKC subtypes^31^. Furthermore, the effect of the phorbol ester PDBu (200 nM), an activator of both cPKCs and nPKCs, and U73122 (10 nM), a phospholipase C inhibitor, were also analysed. The results obtained demonstrated that a variation in P2X7 transcript level exclusively occurred when cells were treated with 10 μM GF109203X, whereas 1 μM GF109203X, PDBu, and U73122 had no effect (see supplementary Fig. S3A online), suggesting that *P2rx7* gene expression is controlled by aPKCs. Atypical isotypes (PKCι/λ and PKCζ) have been reported to be critically involved in cell proliferation and survival, although showing different distribution patterns: while PKCι/λ is restricted to testis, insulin secreting cells and epidermis; PKCζ is widely expressed in all tissues, including neuroblastoma^32^,^33^. In N2a cells, serum deprivation resulted in rapid and transient phosphorylation of PKCζ activation loop (Thr^410^), reaching top levels 15 min after serum starvation (see supplementary Fig. S3B online). In order to identify the downstream target of PKCζ involved in the regulation of *P2rx7* gene expression, Sp1 was the first candidate to examine. Mithramycin A was able to abolish the upregulation of P2X7 transcript induced by GF109203X, suggesting that PKCζ was regulating the transcriptional activity of Sp1 (Fig. 7A). A similar assay was performed in SH-SY5Y human neuroblastoma cells, where GF109203X treatment increase slightly but significantly the expression of P2X7 mRNA, effect that was prevented by mithramycin addition (see supplementary Fig. S1D).

Direct phosphorylation of Sp1 by PKCζ has been reported by several groups, although all of them described the induction of Sp1-dependent gene expression upon phosphorylation by this kinase^28^. Alternatively, PKCζ could be indirectly regulating Sp1 transcriptional activity by hampering PI3K/Akt pathway, since PKCζ has been reported to be a mediator of Akt inactivation^34^. In order to know whether PKCζ is regulating *P2rx7* gene expression by interfering PI3K/Akt pathway, serum-deprived N2a cells were co-treated with GF109203X and inhibitors of PI3K/Akt pathway. As shown in Fig. 7B, either LY294002 or API-1 abolished the potentiatory effect of GF109203X alone, so direct phosphorylation of Sp1 by PKCζ appeared to be unlikely. Western blot and immunofluorescence studies revealed that inhibition of PKCζ significantly increased the nuclear levels of Sp1 in N2a cells, effect that was completely prevented by PI3K inhibition (Fig. 7C,D, respectively). Noticeable, GF109203X treatment also increased Sp1 protein levels in SH-SY5Y human neuroblastoma cells, effect that was completely abolished by PI3K inhibition (see supplementary Fig. S1C). The ability of PKCζ to negatively regulate Akt may imply a hypothetical interaction between PKCζ and Akt that abrogates Akt phosphorylation^35^. In order to evaluate this possibility, Akt phosphorylation was analyses in serum-deprived N2a cells treated with GF109203X. As shown in Fig. 7E, inhibition of PKCζ significantly enhanced Akt phosphorylation induced by serum withdrawal, indicating that PKCζ is negatively regulating Akt activity.

## Discussion

P2X7R is consistently and highly expressed in a wide variety of human cancers, including prostate, cervical and breast cancer, melanoma, colon carcinoma, hematological malignancies and neuroblastic tumors^1^,^4^. The most remarkable property conferred by P2X7R to cancer cells is the ability to survive under limiting growth conditions, such as serum and glucose deprivation, allowing higher proliferation rates and decreased apoptosis^15^. The growth-promoting activity of P2X7R seems to be also related to the stimulation of vascular endothelial growth factor (VEGF) secretion and to the facilitation of extracellular matrix invasion^5^,^8^,^36^,^37^. In neuroblastoma cells, where P2X7R activation enhances proliferation^5^,^6^, serum withdrawal raises P2X7R expression, thus facilitating the maintenance of cancer cells in a proliferative state even in the absence of trophic support^11^,^18^. The objective of this study was to clarify the mechanism by which serum deprivation enhance the expression of P2X7R in neuroblastoma cells. Thus, we have identified the PI3K/Akt/Sp1 axis as the central signaling pathway involved in the increase of P2X7R expression following serum starvation. We took advantage of the N2a cellular model, which is a murine neuroblastoma that express native P2X7Rs and has been widely characterized by our group^10^,^14^,^18^. Moreover, in an effort to extend our findings to a human cellular model, we have also confirmed the main results in SH-SY5Y cell line, a human neuroblastoma that express functional P2X7R^19^. Serum withdrawal augmented P2X7 transcript and protein levels in neuroblastoma cells, effects that were completely prevented by treatment with PI3K inhibitor LY294002. The increase in P2X7R expression elicited by serum deprivation facilitated neuroblastoma cell survival in the absence of trophic support, since the P2X7R antagonists BBG and A740003 exerted a reduction in N2a cell growth comparable to that produce by LY294002. PI3K/Akt pathway is one of the most important signaling cascade in human cancer, playing a relevant role in growth and survival of many tumor types, and being associated to neuroblastoma progression and resistance to chemotherapy^21^,^25^,^38^,^39^. PI3K-dependent activation of Akt is a multistep process involving the recruitment of Akt to the plasma membrane as a result of its binding to phosphatidylinositol 3,4-diphosphate and phosphatidylinositol 3,4,5-triphosphate generated by PI3K activity. Once in the membrane, Akt is phosphorylated on Thr^308^ and Ser^473^ by 3-phosphoinositide-dependent protein kinases (PDKs) named PDK-1 and PDK-2, respectively^40^,^41^. Phosphorylation of Thr^308^ is necessary and sufficient for Akt activation, whereas phosphorylation of Ser^473^ is not sufficient but necessary for maximal activation of Akt^42^. In this work, we have demonstrated that serum deprivation induced a rapid and prolonged phosphorylation of Akt at Thr^308^, which correlates with the upregulation of *P2rx7* gene expression since the Akt inhibitor API-1 practically abolished P2X7 transcript. These evidences point to Akt as the central player in the regulation of P2X7R levels in neuroblastoma cells. More than 12 Akt substrates have been identified in mammalian cells, being GSK3 and mTOR the principal downstream effectors involved in cell growth^24^. Akt-dependent phosphorylation of GSK3, which leads to its inactivation by proteasomal degradation, has been associated with many pathological conditions including cancer^43^. GSK3 is a well-known negative regulator of glycogen synthase, and glycogen is emerging as the energy supply for cancer cells that allows their survival in metabolic stress conditions and hypoxia^44^,^45^. On the other hand, mTOR is a master regulator of protein synthesis that controls cell proliferation and metabolism, and frequently over-activation and dysregulation of the mTOR pathway has been implicated in neuroblastoma pathogenesis^46^. Unexpectedly, our studies demonstrate that neither mTOR nor GSK3 inhibitors disturb the expression of P2X7R in neuroblastoma cells, indicating that non-canonical downstream effectors of Akt, or even directly Akt, should be implicated in the transcriptional regulation of *P2rx7* gene. In a previous study, we reported that Sp1 factor plays a key role in the transcriptional regulation of *P2rx7* gene^18^. The stability, location and transcriptional activity of Sp1 is accurately regulated by post-transcriptional modifications such as phosphorylation, and a significant number of kinases have been found to be involved in the phosphorylation of Sp1 factor, including Akt^28^. Recent studies have demonstrated that Akt phosphorylates Sp1 at residues Ser^42^, Thr^679^ and Ser^698^, resulting in higher protein stability and DNA binding activity of Sp1^47^. Furthermore, Akt is associated with Sp1 phosphorylation in glioblastoma cells and prostate cancer cells, where Sp1 is involved in Akt-mediated induction of VEGF expression that contributes to the growth of tumors by increasing angiogenesis^48^. Here, we demonstrated that serum withdrawal increased Sp1 expression in N2a cells and that the inhibitor of Sp1-mediated transcriptional activation mithramycin abolished the upregulation of *P2rx7* gene expression induced by serum deprivation. Treatment with LY294002 and API-1 significantly reduced nuclear Sp1 levels, suggesting that Sp1 is a downstream effector of PI3K/Akt pathway in neuroblastoma cells. Signaling by PI3K pathway is classically found downstream of tyrosine kinase receptors, which play an important role in survival, growth, and differentiation of many normal and malignant cells. The expression of EGFR in tumors has been correlated with faster progression, poor survival, poor response to therapy, and resistance to cytotoxic agents^49^. The ability of cholesterol depletion to induce ligand-independent activation of EGFR and enhance EGFR phosphorylation has been observed by several groups^50^,^51^,^52^. Apparently, cholesterol depletion is accompanied by the disruption of lipid rafts and the loss of the EGFR from this compartment, which may affect EGFR conformation and function through lipid-based mechanisms^53^,^54^. Subsequent studies have shown that serum withdrawal triggers ligand-independent activation of EGFR in N2a cells, mainly due to high-density lipoprotein removal^29^. Furthermore, treatment of N2a cells with statins, which impair cholesterol biosynthesis and are widely used in the treatment of hyperlipidemia, resulted in phosphorylation of EGFR and downstream kinases such as PI3K and Akt^55^. In this work, we have demonstrated that serum deprivation induced a rapid and prolonged phosphorylation of Akt that was drastically prevented by EGFR inhibition, suggesting that serum withdrawal-dependent activation of EGFR is required to trigger PI3K/Akt signaling cascade and, consequently, to induce Sp1-dependent upregulation of P2X7R in neuroblastoma cells. However, we cannot rule out that other upstream modulators different from growth factor receptors could be activating PI3K in neuroblastoma cells. Indeed, a recent publication from Adinolfi’s group nicely demonstrated that P2X7R triggers PI3K/Akt activation in neuroblastoma cell lines and derived tumors, thus affecting the main downstream effectors implicated in neuroblastoma progression: GSK3β/MYCN and HIFα/VEGF pathways^9^. If this is the case, the present work completes the knowledge about the tumor-promoting role of P2X7R, demonstrating that in circumstances of trophic deprivation this receptor is able to upregulate its own expression via PI3K/Akt pathway in order to facilitate tumor cell proliferation, energy production and migration of cancer cells.

We have also provided evidences that atypical PKCζ is playing a relevant role in the regulation of *P2rx7* gene expression in neuroblastoma cells lacking trophic support. Similarly to Akt, PKCζ is activated by a PI3K-dependent mechanism mediated by PDK-1, which implicates the phosphorylation of Thr^410^ in the activation loop of PKCζ. Following phosphorylation, PKCζ rapidly autophosphorylate on Thr^560^ in the catalytic domain, leading to full activation of the enzyme^56^,^57^. Accordingly, we have observed that serum deprivation elicited a rapid and prolonged phosphorylation on Thr^410^ residue of PKCζ in N2a cells, presumably leading to PKCζ activation. Moreover, disruption of PI3K signaling completely blocked the increased in nuclear levels of Sp1 induced by the PKC inhibitor GF109203X. Next, we faced the question of how PKCζ is regulating Sp1-dependent *P2rx7* gene expression in neuroblastoma cells. Certainly, a direct phosphorylation of Sp1 by PKCζ has been reported by several groups^28^. In 1998, Pal and co-workers showed that PKCζ selectively interacts with Sp1 and phosphorylates its zinc finger domain^58^. Since then a number of studies have confirmed PKCζ-dependent phosphorylation of Sp1. Angiotensin II, which activates PKCζ phosphorylation, stimulates Sp1 phosphorylation and increases Sp1 binding to the platelet-derived growth factor-D promoter^59^. Phosphorylation of Sp1 by PI3K/PKCζ is also critical for trichostatin A-activated luteinizing hormone receptor gene expression in choriocarcinoma cells^60^. In human kidney cells, 1,25-dihydroxyvitamin D3 induces 25-hydroxyvitamin D3 24-hydroxylase gene transcription via PI3K/PKCζ pathway and the phosphorylation of Sp1^61^. In addition, low-density lipoprotein treatment elevated PKCζ phosphorylation and induced PKCζ-dependent phosphorylation of Sp1 in a macrophage cell line, leading to ATP-binding cassette transporter A1 gene expression^62^. All of these studies reported the induction of Sp1-dependent gene expression upon phosphorylation by PKCζ. However, we have demonstrated that PKCζ is downregulating Sp1-dependent expression of *P2rx7* gene in N2a cells, so direct modulation of Sp1 activity by this kinase seems unlikely in our specific cellular context. Furthermore, upregulation of Sp1-dependent *P2rx7* gene expression by GF109203X requires Akt activation, suggesting that PKCζ could be reducing Sp1-dependent transcription by negatively regulating Akt. Strong support for this hypothesis was found from the evidence that PKCζ inhibition raised the phosphorylation state of Akt induced by serum withdrawal. Previous studies have demonstrated that Akt interacts with PKCζ *in vivo* and that PKCζ acts as a negative regulator of Akt^34^. Moreover, in breast cancer cells PKCζ physically and functionally binds to Akt, resulting in the inhibition of Akt phosphorylation at both Thr^308^ and Ser^473^, and leading to a decrease in Akt activity that limits signaling coupled to PI3K/Akt pathway^63^.

**Figure 8:**
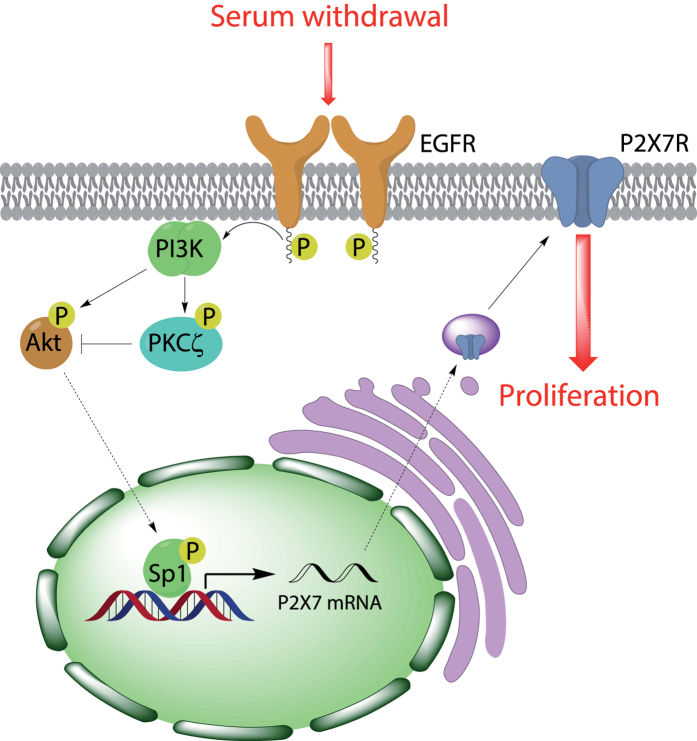
Serum withdrawal upregulates pro-survival P2X7R in neuroblastoma cells. Serum starvation triggers EGF-independent activation of EGFR and, consequently, activation of PI3K/Akt pathway, resulting in Sp1 phosphorylation and induction of *P2rx7* gene expression. Moreover, PKCζ activation reduces Akt phosphorylation, thereby providing a negative feedback loop to regulate Akt activity. Higher levels of P2×7R allow cell proliferation of N2a cells even in the absence of trophic support.

In summary, our study sheds light on the biochemical mechanisms leading to serum withdrawal-induced upregulation of P2X7R expression in neuroblastoma cells, implicating well-known players such as EGFR, PI3K, Akt, PKC and Sp1 in this process (a simplified scheme is represented in Fig. 8). Thus, serum starvation triggers ligand-independent activation of EGFR that leads to activation of PI3K/Akt pathway, resulting in Sp1 phosphorylation that presumably would enhance the stability and DNA binding activity of this transcription factor^47^. Consequently, an induction of *P2rx7* gene expression takes place, increasing the endogenous levels of P2X7 transcript and protein in neuroblastoma cells, and allowing cell proliferation even in the absence of trophic support. Furthermore, atypical PKCζ is also playing a key role in the regulation of *P2rx7* gene expression through the inhibition of Akt activity. Based on elegant preclinical studies reporting a clear oncogenic role of P2X7R in neuroblastoma growth and progression^9^, there are three clinically relevant implications from our *in vitro* data: 1) nutritional factors and cholesterol might play a role in the clinical outcome of neuroblastoma patients through the regulation of P2X7R expression levels; 2) tumors generated by pathological activation of either EGFR or PI3K might be also overexpressing P2X7R; and 3) pharmacological interventions aimed at the signaling cascade outlined in this study might be therapeutically relevant for the development of more effective and less toxic treatments (for instance, combinations of selective inhibitors against EGFR, PI3K/Akt pathway, Sp1 and/or P2X7R).

## Methods

### Antibodies and chemicals

LY294002, API-1, KN93, H89, A740003, and ARL67156 were obtained from Tocris Bioscience (Bristol, UK). GF109203X and U-0126 were supplied by Merck Millipore (Billerica, MA). The tetrazolium dye MTT was from Sigma (St. Louis, MO). The commercial antibodies used in this study were raised against: P2X7R intracellular epitope (PR-004, Alomone Laboratories, Jerusalem, Israel), Sp1 (#07-645, Merck Millipore), phospho-Akt (Thr^308^) (#2965, Cell Signaling, Leiden, The Netherlands), Akt (#9272, Cell Signaling), phospho-PKCζ (Thr^410^) (sc-271962, Santa Cruz Biotechnology, Heidelberg, Germany), PKCζ (sc-7262, Santa Cruz Biotechnology), histone H2B (#2934, Cell Signaling), α-tubulin (T5168, Sigma) and GAPDH (G9545, Sigma). Secondary Alexa Fluor® conjugate antibodies were from Molecular Probes (Madrid, Spain). EGF and mithramycin A were provided by Sigma. All other reagents not specified were routinely supplied by Merck Millipore or Sigma.

### Cell culture and transfection

Murine neuroblastoma cell line N2a (ATCC n° CCL-131) and human neuroblastoma cell line SH-SY5Y (ATCC n° CRL-2266) were grown in DMEM supplemented with 10% FBS, glutamax®, 100 U/ml penicillin, and 100 μg/ml streptomycin (Gibco, Madrid, Spain). All cell cultures were grown at 37 °C in humified atmosphere containing 5% CO_2_. For serum withdrawal experiments, cells were seeded on 6-well plates at a density of 75,000 cels/cm^2^ for 12 hours. Afterwards, complete medium was change for DMEM without serum and cells remained 24, 48 or 72 h, depending on each experiment. Transient transfections of plasmid DNAs were carried out using Lipofectamine™ 2000 (Invitrogen, Madrid, Spain) following the manufacturer’s instructions. Transfection efficiency of N2a cells was ≥90%. Plasmids for overexpression of GSK3 (pcDNA3-GSK3) and constitutively active GSK3-S9A (pcDNA3-GSK3S9A) were provided by Dr. J.J. Lucas laboratory, and have been previously checked in our cellular model^26^.

### RT-PCR and quantitative real-time PCR

Total RNA was extracted from cultured neuroblastoma cells using Speedtools total RNA extraction kit (Biotools, Madrid, Spain), following the manufacturer’s instructions. After digestion with TURBO DNase (Ambion), total RNA was quantified and reversed transcribed using M-MLV reverse transcriptase, 6 μg of random primers and 350 μM dNTPs (Invitrogen). Quantitative real-time PCR reactions were carried out using LuminoCt^®^ qPCR readymix (Sigma), 5 μL of the RT product, and gene-specific primers and TaqMan MGB probes for mouse P2X7, Sp1 and GAPDH (Applied Biosystems, Madrid, Spain). Fast thermal cycling was performed using a StepOnePlus™ Real-Time PCR System (Applied Biosystems) as follows: denaturation, one cycle of 95 °C for 20 sec, followed by 40 cycles each of 95 °C for 1 sec and 60 °C for 20 sec. The results were normalized as indicated by parallel amplification of the endogenous control GAPDH.

### Western blotting

Protein extracts from neuroblastoma cells were obtained by lysis at 4 °C in extraction buffer containing 50 mM Tris/HCl, 150 mM NaCl, 1% Nonidet P40 and Complete™ Protease Inhibitor Cocktail Tablets (Roche Diagnostics, Barcelona, Spain), pH 7.4. Nuclear fraction of N2a cells (400 μl) was obtained with ProteoExtract Subcellular Proteome Extraction Kit (Merck Millipore), according to manufacturer instructions. Protein extracts (30 μg) were electrophoresed on 8% Tris-Glycine SDS-PAGE gel and transferred to nitrocellulose membranes (Amersham GE, Barcelona, Spain) saturated for 1 h at room temperature with 5% non-fat dried milk. Primary antibodies used were: anti-P2X7R (1:1000), anti-Sp1 (1:1000), anti-phospho-Akt (Thr^308^) (1:1000), anti-Akt (1:1000), anti-phospho-PKCζ (Thr^410^) (1:500), anti-PKCζ (1:500), anti-histone H2B (1:1000), and anti-GAPDH (1:5000, 37 kDa). Proteins were visualized by enhanced chemoluminescence detection (Perkin Elmer, Houston, TX). Images were captured with ImageQuant LAS 500 (Amersham GE) and analysed using ImageJ 1.47t software (NIH, Bethesda, MD).

### Immunofluorescence studies

N2a cells cultured on coverslips placed in 35 mm dishes (30,000 cels/cm^2^) were fixed in 4% PFA for 15 min. After washing with PBS, cells were permeabilized with 0.1% Triton X-100 and blocked with 5% goat serum and 10% FBS for 1 h at room temperature. After washing with 3% BSA in PBS, cells were incubated for 1 h with primary antibodies against P2X7R (1:200), Sp1 (1:200) and/or α-tubulin (1:500). Subsequently, cells were washed with PBS and incubated for 1 h with appropriate secondary Alexa Fluor® conjugate antibodies (1:400) and nuclei were counterstained with DAPI. Coverslips were mounted using Prolong^®^ gold antifade reagent (Molecular Probes). Confocal images were acquired with a TCS SPE microscope from Leica Microsystems with a 63× Apochromat NA = 1.3 oil objective lens (Wetzlar, Germany) and analyzed with ImageJ software.

### Cell proliferation assays

N2a cells were plated in 6-well plates (20,000 cels/cm^2^) and maintained for 2 h in DMEM containing 10% FBS. Then, cells were starved by replacing the medium with serum-free DMEM containing LY294002, ARL67156, BBG and/or A740003. After 48 h, cell proliferation was assessed using the colorimetric MTT assay. The tetrazolium dye MTT 3-(4,5-dimethylthiazol-2-yl)-2,5-diphenyltetrazolium bromide was added to cells to a final concentration of 0.5 mg/mL, and maintained for 2 h at 37 °C in humified atmosphere containing 5% CO_2_. Then, an equal volume of MTT solubilization solution (10% Triton X-100 plus 0.1 N HCl in anhydrous isopropanol) was added, following a brief incubation of 30 min at room temperature with orbital shaking. The samples were collected and measured spectrophotometrically at 570 nm. Values were normalized to those obtained from untreated control cells, set as 100% proliferation rate.

### Statistical analysis

Data were analyzed using one way-ANOVA with the Dunnett’s post hoc test or unpaired t-test for two-group comparisons. For multiple comparisons, one-way ANOVA analyses were corrected with Sidak’s post hoc test (Graph Pad Prism 5, Graph Pad Software Inc., San Diego, CA). Statistical analyses used are indicated in figure legends. Data are expressed as mean ± s.e.m. of a minimum of three independent experiments in duplicate. A value of *P *≤ 0.05 was considered statistically significant.

## Additional Information

**How to cite this article**: Gómez-Villafuertes, R. *et al.* PI3K/Akt signaling pathway triggers P2X7 receptor expression as a pro-survival factor of neuroblastoma cells under limiting growth conditions. *Sci. Rep.* **5**, 18417; doi: 10.1038/srep18417 (2015).

## Supplementary Material

Supplementary Information
