# Supplementary material for: PI3K/Akt signaling pathway triggers P2X7 receptor expression as a pro-survival factor of neuroblastoma cells under limiting growth conditions
Source: Sci Rep. 2015 Dec 21;5:18417. doi: 10.1038/srep18417 (PMC4685307; doi:10.1038/srep18417)
Supplement: Supplementary Information [file srep18417-s1.pdf]

PI3K/Akt signaling pathway triggers P2X7 receptor expression as a pro-survival factor of neuroblastoma cells under limiting growth conditions

Rosa Gómez-Villafuertes[1,2*†], Paula García-Huerta[1,2*], Juan Ignacio Díaz-Hernández[1,2], Mª Teresa Miras-Portugal[1,2].

Fig. S1. Effect of serum deprivation in P2X7R expression in SH-SY5Y human neuroblastoma cell line. (A) Changes in P2X7 transcript levels in SH-SY5Y cell line cultured either in standard culture medium (FBS) for 24 h or in serum free medium (SF) for the indicated time periods. Total RNA was extracted and P2X7 mRNA was quantified by Q-PCR as described in Methods. GAPDH was used as a control for differences in cDNA input. Results are mean ± s.e.m. of three independent experiments in triplicate; *P<0.05, **P<0.01, ***P<0.001 vs FBS (ANOVA with the Dunnett's post hoc test). (B) SH-SY5Y cells were incubated in FBS medium for 24 h or in SF medium in absence (control) or presence of either LY294002 (50 μM, PI3K inhibitor) or API-1 (10 μM, Akt inhibitor) in SF medium for 24 h. Total RNA was extracted and P2X7 mRNA was quantified by Q-PCR, using GADPH as housekeeping gene. Normalized P2X7 transcript levels in cells cultured in FBS was set as 100%. Results are mean ± s.e.m. of three independent experiments in triplicate. *P<0.05, **P<0.01 vs control (ANOVA with the Dunnett's post hoc test). (C) Immunoblotting depicting the presence of endogenous Sp1 in whole-cell lysates from control and LY294002- and/or GF109203X-treated SH-SY5Y cells cultured in SF medium for 24h. GAPDH was used as an internal loading control. Histogram represents levels of Sp1 protein in control cells compared to treated cells, obtained by densitometry and normalization to GAPDH. The values represent mean ± s.e.m. of three independent experiments in duplicate. *P<0.05, **P<0.01 vs control (ANOVA with the Dunnett's post hoc test); ### P<0.001 (ANOVA with the Sidak's post hoc test). (D) Changes in P2X7 transcript levels in serum-deprived SH-SY5Y cells cultured for 24h in absence or presence of 10 μM GF109203X and/or 300 nM mithramycin A (Sp1 inhibitor). GADPH was used as housekeeping gene. Results are mean ± s.e.m. of three independent experiments in triplicate; *P<0.05 vs control (ANOVA with the Dunnett's post hoc test); ### P<0.001 (ANOVA with the Sidak's post hoc test).

Fig. S2. Upregulation of P2X7R expression in serum deprived neuroblastoma cells is independent of both GSK3 and mTOR. (A) N2a cells were incubated in absence or presence of either SB216763 (5 μM, GSK3 inhibitor) or rapamycin (2.5 nM, mTOR inhibitor) in SF medium for 24 h. Total RNA was extracted and P2X7 mRNA was quantified by Q-PCR, using GADPH as a housekeeping gene. Normalized P2X7 transcript levels in cells cultured in FBS medium was set as 100%. Results are mean ± s.e.m. of three independent experiments in triplicate. (B) N2a cells were transiently transfected with empty vector (control), wild-type GSK3β (wtGSK3) or a constitutively active GSK3β mutant ($GSK3^{S9A}$). After 24 h in SF medium total RNA was extracted and P2X7 mRNA was quantified by Q-PCR, using GADPH as a housekeeping gene. Normalized P2X7 expression is expressed as a percentage compared to control cells. Results are mean ± s.e.m. of three independent experiments in triplicate. (C) Immunoblotting showing the presence of endogenous P2X7R in N2a cells transiently transfected with empty vector (control), wild-type GSK3β (wtGSK3) or a constitutively active GSK3β mutant ($GSK3^{S9A}$) and cultured in the absence of serum for 24 h. Whole-cell lysates were analyzed by western blotting with anti-P2X7R antibody. GAPDH was used as an internal loading control. Histogram represents levels of P2X7 protein in control cells compared to cells transfected with either wtGSK3 or $GSK3^{S9A}$, and were obtained by densitometry and normalization to GAPDH expression. Values are mean ± s.e.m. of three independent experiments in triplicate.

Fig. S3. Implication of atypical PKCζ in the regulation of P2X7R expression in neuroblastoma cells after serum withdrawal. (A) Serum-deprived N2a cells were incubated in absence (control) or presence of 1 µM GF109203X (that only inhibits cPKCs and nPKCs), 10 µM GF109203X, (pan PKC inhibitor), 200 nM PDBu (activator of cPKCs and nPKCs), and 10 nM U73122 (phospholipase C inhibitor) for 24 h. Then, total RNA was extracted and P2X7 mRNA was quantified by Q-PCR, using GADPH as a housekeeping gene. Normalized P2X7 transcript levels in control cells was set as 100%. Results are mean ± s.e.m. of three independent experiments in triplicate. ***$P \leq 0.001$ *vs* control (ANOVA with the Dunnett's post hoc test); $^{\#\#}P<0.01$ (ANOVA with the Sidak's post hoc test). (B) Changes in PKCζ phosphorylation state in N2a cells cultured in SF medium for the indicated time points. Whole-cell lysates were analyzed by western blotting with either anti-phospho PKCζ (Thr^{410}) antibody or total PKCζ. Histogram represents relative levels of phospho-PKCζ (pPKCζ) in each case during the whole detection period, and were obtained by densitometry and normalization to total PKCζ levels. The values represent mean ± s.e.m. of three independent experiments in triplicate. **$P \leq 0.01$, ***$P \leq 0.001$ *vs* time=0.

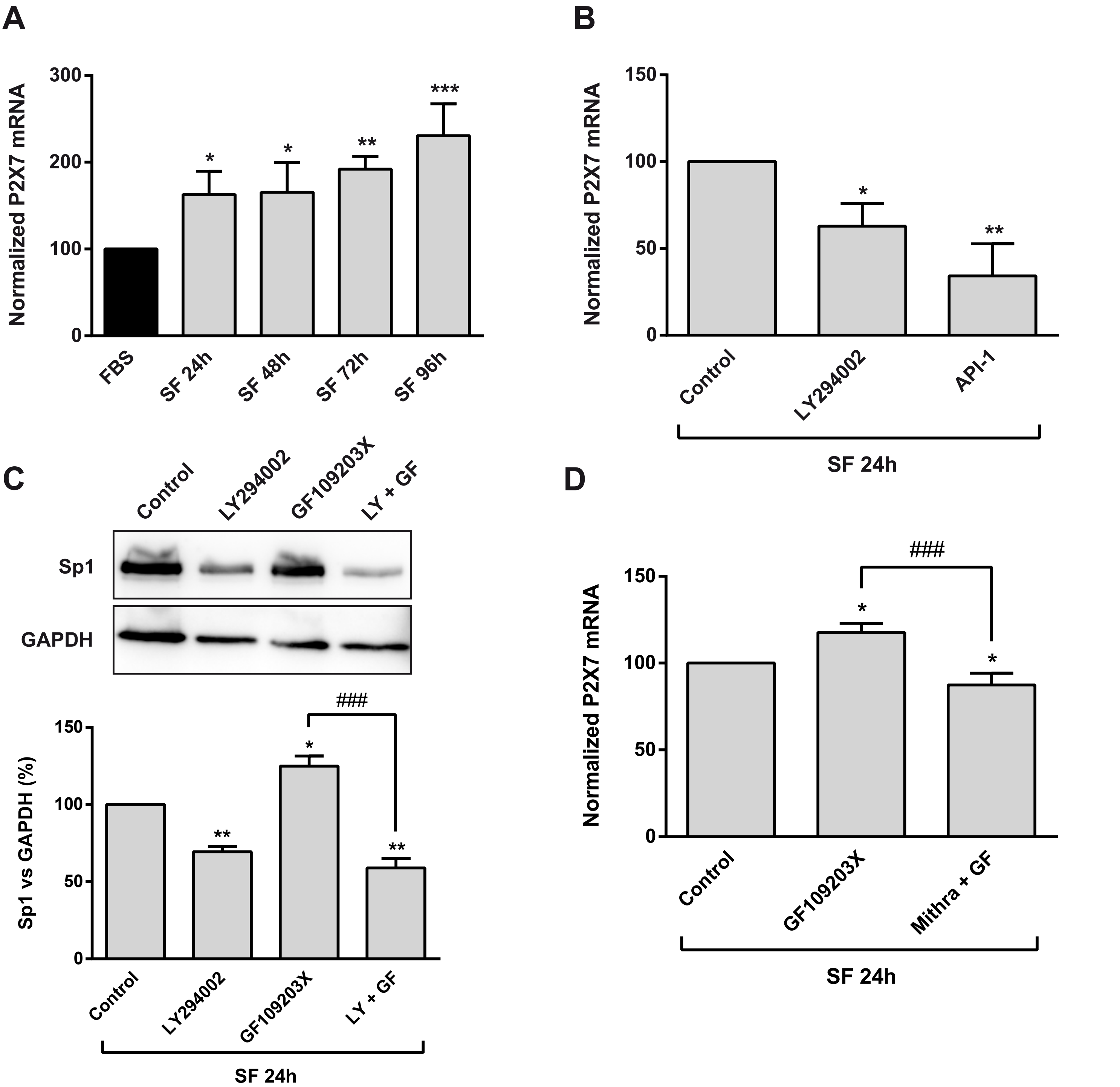

Fig. S1. Effect of serum deprivation in P2X7R expression in SH-SY5Y human neuroblastoma cell line. (A) Changes in P2X7 transcript levels in SH-SY5Y cell line cultured either in standard culture medium (FBS) for 24 h or in serum free medium (SF) for the indicated time periods. Total RNA was extracted and P2X7 mRNA was quantified by Q-PCR as described in Methods. GAPDH was used as a control for differences in cDNA input. Results are mean ± s.e.m. of three independent experiments in triplicate; *P<0.05, **P<0.01, ***P<0.001 vs FBS (ANOVA with the Dunnett's post hoc test). (B) SH-SY5Y cells were incubated in FBS medium for 24 h or in SF medium in absence (control) or presence of either LY294002 (50 μM, PI3K inhibitor) or API-1 (10 μM, Akt inhibitor) in SF medium for 24 h. Total RNA was extracted and P2X7 mRNA was quantified by Q-PCR, using GADPH as housekeeping gene. Normalized P2X7 transcript levels in cells cultured in FBS was set as 100%. Results are mean ± s.e.m. of three independent experiments in triplicate. *P<0.05, **P<0.01 vs control (ANOVA with the Dunnett's post hoc test). (C) Immunoblotting depicting the presence of endogenous Sp1 in whole-cell lysates from control and LY294002- and/or GF109203X-treated SH-SY5Y cells cultured in SF medium for 24h. GAPDH was used as an internal loading control. Histogram represents levels of Sp1 protein in control cells compared to treated cells, obtained by densitometry and normalization to GAPDH. The values represent mean ± s.e.m. of three independent experiments in duplicate. *P<0.05, **P<0.01 vs control (ANOVA with the Dunnett's post hoc test); ### P<0.001 (ANOVA with the Sidak's post hoc test). (D) Changes in P2X7 transcript levels in serum-deprived SH-SY5Y cells cultured for 24h in absence or presence of 10 μM GF109203X and/or 300 nM mithramycin A (Sp1 inhibitor). GADPH was used as housekeeping gene. Results are mean ± s.e.m. of three independent experiments in triplicate; *P<0.05 vs control (ANOVA with the Dunnett's post hoc test); ### P<0.001 (ANOVA with the Sidak's post hoc test).

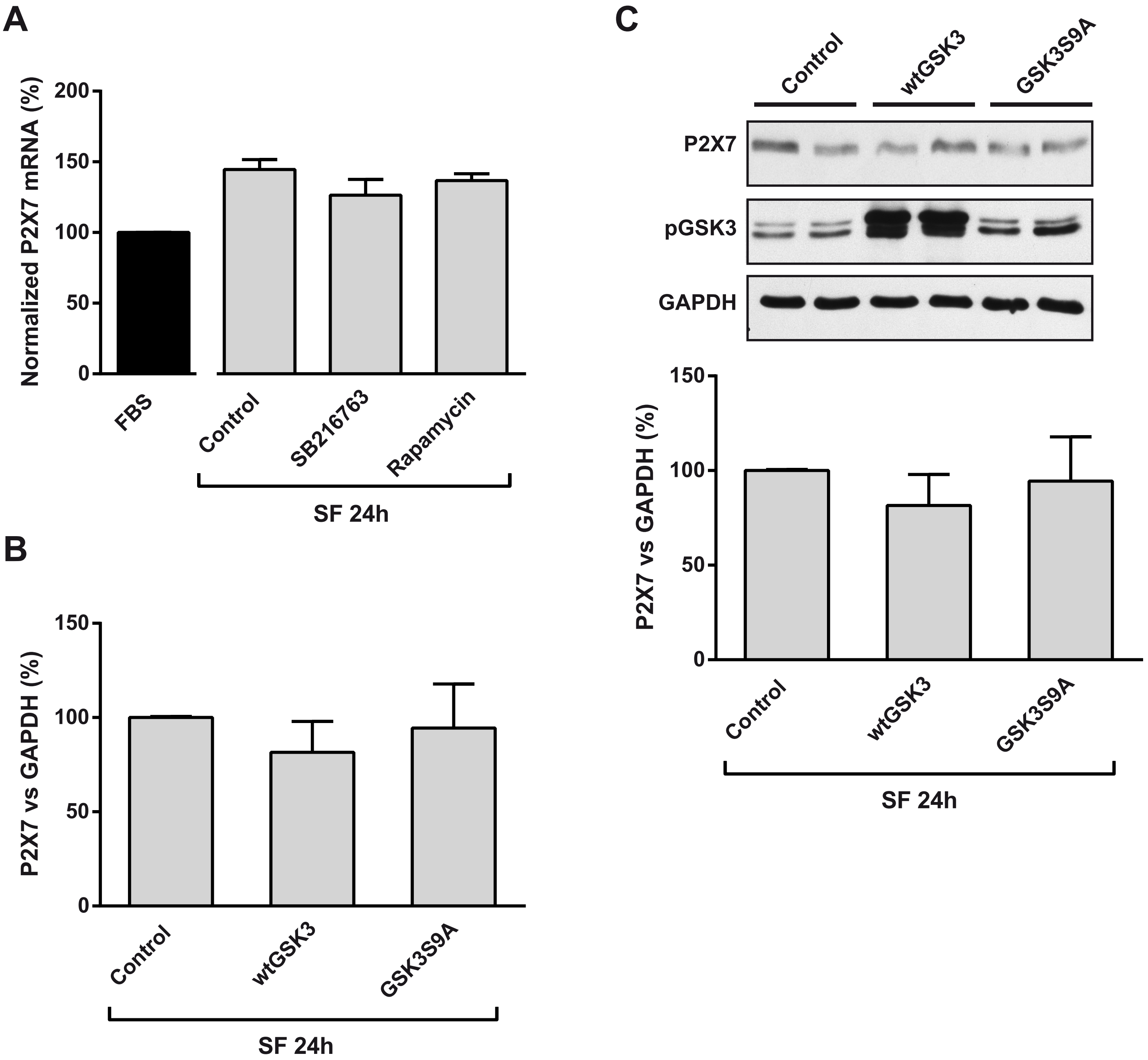

Fig. S2. Upregulation of P2X7R expression in serum deprived neuroblastoma cells is independent of both GSK3 and mTOR. (A) N2a cells were incubated in absence or presence of either SB216763 (5 μM, GSK3 inhibitor) or rapamycin (2.5 nM, mTOR inhibitor) in SF medium for 24 h. Total RNA was extracted and P2X7 mRNA was quantified by Q-PCR, using GADPH as a housekeeping gene. Normalized P2X7 transcript levels in cells cultured in FBS medium was set as 100%. Results are mean ± s.e.m. of three independent experiments in triplicate. (B) N2a cells were transiently transfected with empty vector (control), wild-type GSK3β (wtGSK3) or a constitutively active GSK3β mutant (GSK3S9A). After 24 h in SF medium total RNA was extracted and P2X7 mRNA was quantified by Q-PCR, using GADPH as a housekeeping gene. Normalized P2X7 expression is expressed as a percentage compared to control cells. Results are mean ± s.e.m. of three independent experiments in triplicate. (C) Immunoblotting showing the presence of endogenous P2X7R in N2a cells transiently transfected with empty vector (control), wild-type GSK3β (wtGSK3) or a constitutively active GSK3β mutant (GSK3S9A) and cultured in the absence of serum for 24 h. Whole-cell lysates were analyzed by western blotting with anti-P2X7R antibody. GAPDH was used as an internal loading control. Histogram represents levels of P2X7 protein in control cells compared to cells transfected with either wtGSK3 or GSK3S9A, and were obtained by densitometry and normalization to GAPDH expression. Values are mean ± s.e.m. of three independent experiments in triplicate.

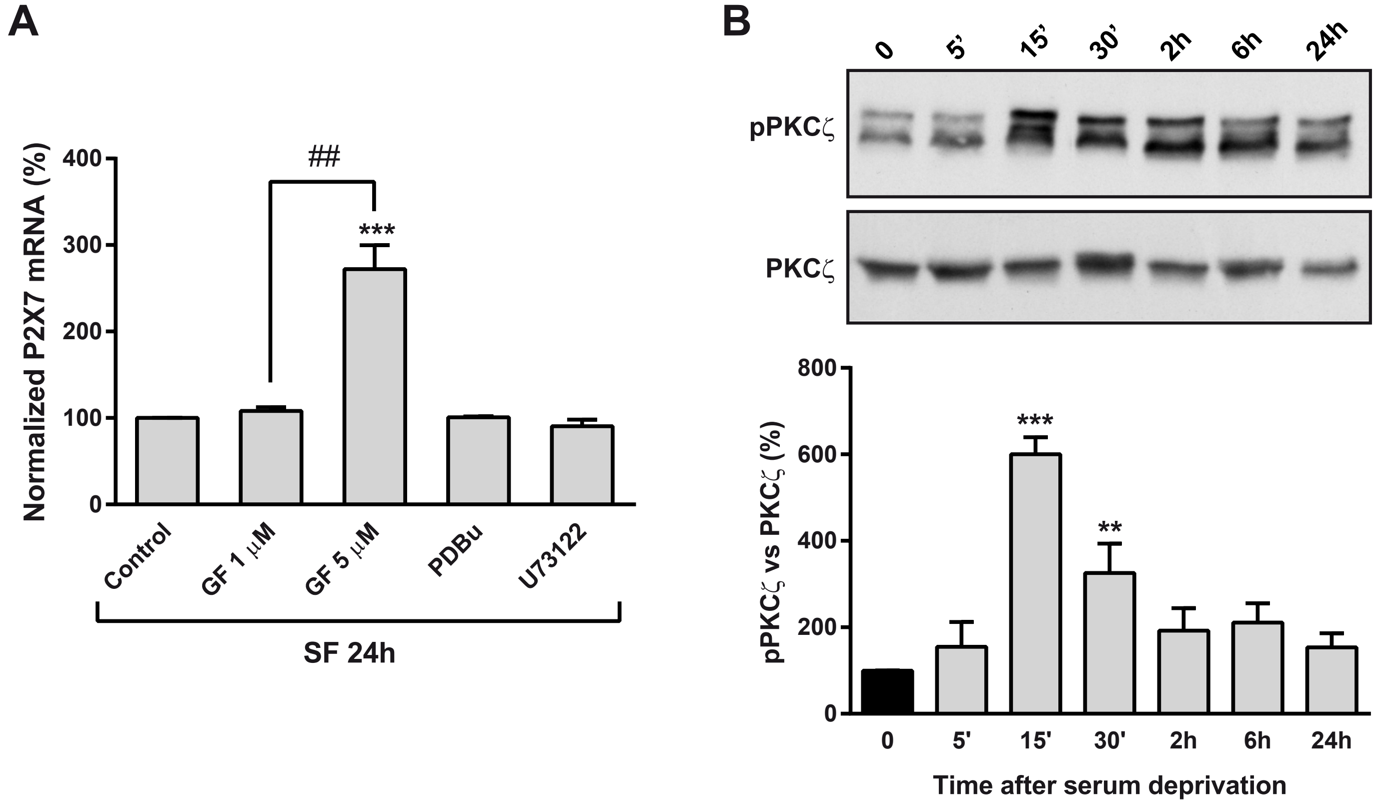

Fig. S3. Implication of atypical PKCζ in the regulation of P2X7R expression in neuroblastoma cells after serum withdrawal. (A) Serum-deprived N2a cells were incubated in absence (control) or presence of 1 μM GF109203X (that only inhibits cPKCs and nPKCs), 10 μM GF109203X, (pan PKC inhibitor), 200 nM PDBu (activator of cPKCs and nPKCs), and 10 nM U73122 (phospholipase C inhibitor) for 24 h. Then, total RNA was extracted and P2X7 mRNA was quantified by Q-PCR, using GADPH as a housekeeping gene. Normalized P2X7 transcript levels in control cells was set as 100%. Results are mean ± s.e.m. of three independent experiments in triplicate. ***P>0.001 vs control (ANOVA with the Dunnett's post hoc test); ##P<0.01 (ANOVA with the Sidak's post hoc test). (B) Changes in PKCζ phosphorylation state in N2a cells cultured in SF medium for the indicated time points. Whole-cell lysates were analyzed by western blotting with either anti-phospho PKCζ (Thr410) antibody or total PKCζ. Histogram represents relative levels of phospho-PKCζ (pPKCζ) in each case during the whole detection period, and were obtained by densitometry and normalization to total PKCζ levels. The values represent mean ± s.e.m. of three independent experiments in triplicate. **P>0.01, ***P>0.001 vs time=0.
